# Characterization of Bio-Inspired Electro-Conductive Soy Protein Films

**DOI:** 10.3390/polym13030416

**Published:** 2021-01-28

**Authors:** Pedro Guerrero, Tania Garrido, Itxaso Garcia-Orue, Edorta Santos-Vizcaino, Manoli Igartua, Rosa Maria Hernandez, Koro de la Caba

**Affiliations:** 1 BIOMAT Research Group, University of the Basque Country (UPV/EHU), Escuela de Ingeniería de Gipuzkoa, Plaza de Europa 1, 20018 Donostia-San Sebastián, Spain; tania.garrido@ehu.eus; 2 BCMaterials, Basque Center for Materials, Applications and Nanostructures, UPV/EHU Science Park, 48940 Leioa, Spain; 3 NanoBioCel Group, Laboratory of Pharmaceutics, School of Pharmacy, University of the Basque Country (UPV/EHU), Paseo de la Universidad 7, 01006 Vitoria-Gasteiz, Spain; itxaso.garcia@ehu.eus (I.G.-O.); edorta.santos@ehu.eus (E.S.-V.); manoli.igartua@ehu.eus (M.I.); rosa.hernandez@ehu.es (R.M.H.); 4 Biomedical Research Networking Centre in Bioengineering, Biomaterials and Nanomedicine (CIBER-BBN), 01006 Vitoria-Gasteiz, Spain

**Keywords:** soy protein, film, semiconductor, biomaterial

## Abstract

Protein-based conductive materials are gaining attention as alternative components of electronic devices for value-added applications. In this regard, soy protein isolate (SPI) was processed by extrusion in order to obtain SPI pellets, subsequently molded into SPI films by hot pressing, resulting in homogeneous and transparent films, as shown by scanning electron microscopy and UV-vis spectroscopy analyses, respectively. During processing, SPI denatured and refolded through intermolecular interactions with glycerol, causing a major exposition of tryptophan residues and fluorescence emission, affecting charge distribution and electron transport properties. Regarding electrical conductivity, the value found (9.889 × 10^−4^ S/m) is characteristic of electrical semiconductors, such as silicon, and higher than that found for other natural polymers. Additionally, the behavior of the films in contact with water was analyzed, indicating a controlled swelling and a hydrolytic surface, which is of great relevance for cell adhesion and spreading. In fact, cytotoxicity studies showed that the developed SPI films were biocompatible, according to the guidelines for the biological evaluation of medical devices. Therefore, these SPI films are uniquely suited as bioelectronics because they conduct both ionic and electronic currents, which is not accessible for the traditional metallic conductors.

## 1. Introduction

Conventional electronic devices include various organic/inorganic materials, which are non-biodegradable, or even toxic, and lead to the accumulation of electronic wastes, causing serious ecological pollution [1]. In this context, the development of functional materials has shifted from petrochemical feedstock to natural, renewable and more sustainable resources [2,3]. Owing to their biodegradability and biocompatibility, biopolymer-based conductive materials are arising significant interest in researchers, from both academic and industrial fields, and have emerged as promising candidates for the production of multifunctional electro-conductive components for advanced applications, such as strain sensors, wearable devices, and portable electronic equipment (electrical devices) [4]. In this regard, energy harvesting by nature-driven biodegradable and biocompatible materials, which respond to biomechanical activities, is receiving great attention to develop an alternative energy source for next-generation portable biomedical devices [5,6,7]. Some bio-based materials, such as fish scales [8], cellulose [9], or spider silk [10], have been studied for green energy harvesting. However, the products developed from these raw materials were not completely bio-based materials, since they were chemically treated or/and mixed with other non-bio-based materials.

In order to overcome the above-mentioned drawbacks, the use of proteins can become an attractive alternative for eco-friendly devices. In particular, soy protein isolate (SPI) is a natural, abundant, and available protein, constituted of 18 amino acid residues. This amino acid composition affects the protein structure and, thus, the properties of the products developed [11,12]. In this regard, Las Heras and co-workers [13] determined that SPI was rich in glutamic and aspartic acids, leucine, serine, glycine, alanine and proline amino acids, with cysteine and methionine as the principal sulphur-containing amino acids. In fact, cysteine presents the ability to form inter- and intra-chain disulfide bonds, which play a crucial role in protein-folding pathways and, thus, in protein structure. Furthermore, SPI exhibits a variety of peptides that promote migration and cell proliferation, key factors for tissue regeneration applications [14]; among them, lunasin owns RGD-like sequences required to promote stable cell adhesion [15]. In this regard, the use of SPI films as electronic components can avoid biocompatibility concerns that are present with conventional electronic components based on synthetic polymers, and may allow easier integration into biological systems. Some recent researches [16,17] have shown that natural and nature-inspired materials can be used not only to create organic devices with state-of-the-art performance but also to provide conceptual clues about the molecular design of organic semiconductors. Therefore, understanding interactions in SPI films and their effect on protein structure is essential.

Processing methods also affect the protein structure and, thus, determine the properties of the final product. It is well known that thermomechanical processes, such as extrusion and compression, induce dissociation, denaturation, and aggregation of soy protein subunits [18,19,20]. The protein is disassembled and then, reassembled by disulfide bonds and non-covalent interactions, such as hydrogen bonding, leading to the formation of stable structures. Breaking intermolecular linkages that stabilize the protein in their native state and the orientation and reconstructing the chains with the formation of new intermolecular linkages stabilize the three-dimensional network formed. Additionally, extrusion and compression are versatile and well-established processes used to manufacture polymeric materials, and these thermomechanical processes are relatively inexpensive and amenable to industrial scale-up. Therefore, in this work extrusion was employed to obtain SPI pellets, converted into films by compression molding. Afterward, the aim of this work was focused on the study of the relationship among processing, structure, and properties of SPI films, including optical, mechanical, electrical and biological properties.

## 2. Materials and Methods 

### 2.1. Materials

Soy protein isolate (SPI), PROFAM 974, with 90% protein on a dry basis, was supplied by ADM Protein Specialties Division (Amsterdam, The Netherlands). SPI has 5% moisture, 4% fat, and 5% ash and its isoelectric point is 4.6. Glycerol, with a purity of 99.01% (Panreac, Barcelona, Spain), was used as plasticizer.

### 2.2. Pellets Preparation

SPI and glycerol were mixed in a Stephan UMC 5 mixer for 5 min at 1500 rpm in order to obtain a good blend. Blends were prepared with 80 wt% SPI and 20 wt% glycerol on total mass. These blends were added into the feed hopper of a twin-screw extruder and mixed with water in the extruder barrel. The MPF 19/25 APV Baker extruder used in this study had 19 mm diameter-barrel and a length/barrel diameter ratio of 25:1. Barrel temperatures were set at 70 °C, 80 °C, 95 °C, and 100 °C for the four zones from input to output, and die temperature was set at 100 °C, based on previous work [21]. Water was pumped directly into the extruder barrel at a constant speed of 250 rpm using a peristaltic 504U MK pump (Watson Marlow Ltd., Rommerskirchen, Germany). All trials were carried out using a water speed of 3.0 g/min (0.18 kg/h). The feed rate of the extruder was adjusted to 1 kg/h and a single die of 3 mm diameter was used, giving a throughput per unit area of 0.141 kg/h mm^2^. Extrusion process variables were measured; in particular, the specific mechanical energy (SME), the amount of work input from the driver motor into the extruded material.

### 2.3. Film Preparation

The pellets obtained by extrusion were placed between two aluminum sheets using a caver laboratory press (Specac, Madrid, Spain), previously heated at 120 °C. The pellets were pressed at 80 bar for 2 min to obtain compression-molded films. All samples were conditioned in an ACS Sunrise 700 V bio-chamber at 25 °C and 50% relative humidity for 48 h before testing. 

### 2.4. Film Characterization

#### 2.4.1. Differential Scanning Calorimetry (DSC) and Thermo-Gravimetric Analysis (TGA)

DSC experiments were performed using a Mettler Toledo DSC 822 (Mettler Toledo, Barcelona, Spain). Around 4 mg of sample was heated from −50 °C to 250 °C at a rate of 10 °C/min under nitrogen atmosphere to avoid oxidation reactions. Sealed aluminum pans were used to prevent mass loss during the experiment. 

TGA was carried out using a Mettler Toledo SDTA 851 equipment (Mettler Toledo, Barcelona, Spain). All specimens were heated from room temperature to 800 °C at a heating rate of 10 °C/min under nitrogen atmosphere (10 mL/min) to avoid thermo-oxidative reactions.

#### 2.4.2. Fourier Transform Infrared (FTIR) Spectroscopy

Attenuated total reflectance Fourier transform infrared (ATR-FTIR) spectroscopy was used to identify the characteristic functional groups of SPI films. Measurements were performed with a Nicolet Nexus FTIR spectrometer (ThermoFisher, Madrid, Spain) equipped with an MKII Golden Gate accessory (Specac) with a diamond crystal as ATR element at a nominal incidence angle of 45° with a ZnSe lens. Measurements were recorded in the 4000–750 cm^−1^ region, using 32 scans at a resolution of 4 cm^−1^. All spectra were smoothed using the Savitzky–Golay function. Second-derivative spectra of the amide region were used at peak position guides for the curve fitting procedure, using OriginPro 2019b software.

#### 2.4.3. Ultraviolet-Visible (UV-vis) and Fluorescence Spectroscopies

The spectrophotometer UV-vis-Jasco (Model V-630) (Jasco, Madrid, Spain), coupled to solid sample support, was used to determine light barrier properties of films. Light absorption was measured at wavelengths from 200 nm to 800 nm. 

Fluorescence emission measurements were performed with the spectrophotometer (Horiba, Madrid, Spain) of Photon Technology International (PTI) coupled to a solid sample support. SPI films were excited at 370 nm and emission spectra were recorded from 385 nm to 600 nm, using FeliX32 software.

#### 2.4.4. X-ray Diffraction (XRD) and X-ray Photoelectron Spectroscopy (XPS)

XRD was performed with a diffraction unit PANalytical Xpert PRO (Malvern, Madrid, Spain), operating at 40 kV and 40 mA. The radiation was generated from Cu-Kα (λ = 1.5418 Å) source and the diffraction data were collected from 2θ values from 2° to 90°, where θ is the angle of incidence of the X-ray beam on the sample. All spectra were smoothed using the Savitzky–Golay function. Second-derivative of XRD spectra were used at peak position guides for the curve fitting procedure, using OriginPro 2019b software. 

XPS was performed in a SPECS spectrometer (SPECS, Barcelona, Spain) using a monochromatic radiation equipped with Al Kα (1486.6 eV). The binding energy was calibrated by Ag 3d5/2 peak at 368.28 eV. All spectra were recorded at 90° take-off angle. Survey spectra were recorded with 1.0 eV step and 40.0 eV analyzer pass energy and the high-resolution regions with 0.1 eV step and 20 eV pass energy. All core level spectra were referred to the C 1s peak at 284.6 eV. Spectra were analyzed using the CasaXPS 2.3.19PR 1.0 software, and peak areas were quantified with a Gaussian–Lorentzian fitting procedure.

#### 2.4.5. Scanning Electron Microscopy (SEM)

Morphology of the film cross-section was visualized using a scanning electron microscope S-4800 (Hitachi, Madrid, Spain). The samples were mounted on a metal stub with double-side adhesive tape and coated under vacuum with gold (JFC-1100) in an argon atmosphere prior to observation. All samples were examined using an accelerating voltage of 15 kV.

#### 2.4.6. Mechanical Testing

Tensile strength (TS), and elongation at break (EB) were measured according to ASTM D638-03 [22], using Instron 5967 electromechanical testing system with a tensile load cell of 500 N. Five specimens for each film were cut into bone-shaped samples of 4.75 mm × 22.25 mm, the film thickness was 0.626 mm, and the crosshead rate was 5 mm/min.

#### 2.4.7. Electrical Conductivity

**Figure 1:**
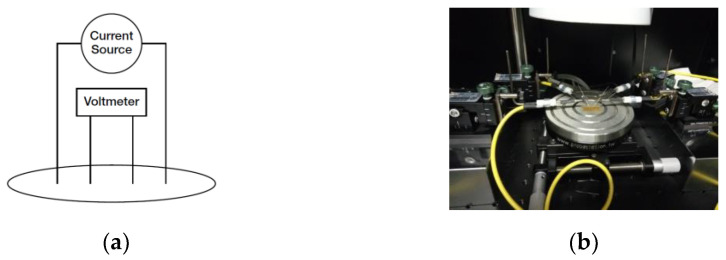
(**a**) Four-point collinear probe resistivity configuration and (**b**) photograph of the home-built dispositive used for the conductivity measurement.

Electrical properties of films were measured by a Keithley 4200-SCS (Mouser, Barcelona, Spain) equipment for semiconductors analysis in a Faraday cage at room temperature with low-noise triax cables and results were analyzed with KITE Software (Keithley Interactive Test Environment 9.0). Two point measurements were carried performing linear scans from −20 V to 20 V in order to obtain intensity vs. voltage curves. The conductivity (resistivity) of SPI films was measured by using a four-point collinear probe (Figure 1). Four electrodes were used in order to minimize any measurement error due to the contact resistance. The films were placed in contact with copper sheets, adhered in turn to polycarbonate plates. The distance between the electrodes was 3 mm and the dimensions of the samples were 2 × 2 cm^2^ section and 0.626 mm height.

#### 2.4.8. Water Uptake

First, dry samples of the films were cut into discs of 8 mm in diameter. Then, they were weighed and immersed in 1 mL of phosphate buffer solution (PBS) at 37 °C for determined periods of time. Samples were collected at specific times, the excess water was wiped out with a filter paper and the wet discs weighed. Water uptake (WU) was calculated as:

(1) $$WU(\%)=\frac{W-W_{0}}{W_{0}}\times 100$$

where *W*_0_ is the weight of the dry discs at the beginning of the study and *W* is the weight of the wet discs at every time point.

#### 2.4.9. Water Contact Angle

Contact angle measurements were carried out by the sessile drop technique using a Dataphysics OCA 20 system (Aries, Madrid, Spain). Samples were laid on a movable sample stage and about 3 μL of distilled water was placed onto the film surface to estimate the hydrophobic character. The contact angle images were recorded on the sample, using SCA20 software. Five measurements were made at room temperature.

#### 2.4.10. Degradation Analyses

To assess the degradation of the films in an aqueous environment, samples were cut into discs of 8 mm in diameter, weighed and immersed into PBS at 37 °C. At determined time points, films were collected, washed with milliQ water and freeze-dried. Dry samples were weighed and the percentage of the remaining weight (RM) was calculated using the following equation:

(2) $$RM\;(\%)=\frac{final\;weight}{initial\;weight}\times 100$$

Enzymatic degradation was assessed by immersing discs of 8 mm in diameter into 1 mL of a 0.5 mg/mL collagenase H solution (Sigma-Aldrich, Saint Louise, MO, USA). Samples were incubated at 37 °C for 1, 4 and 7 days. At those time points, discs were collected, washed with milliQ water, freeze-dried and weighed. RM values were calculated as expressed by Equation (2).

Cellular degradation was evaluated using L-929 fibroblasts (ATCC, Manassas, VA, USA). Cells were cultured in Eagle’s Minimum Essential Medium (EMEM, ATCC, Manassas, VA, USA) supplemented with 1% (*v*/*v*) penicillin-streptomycin and 10% (*v*/*v*) inactivated Horse Serum at 37 °C in a humidified incubator with a 5% CO_2_ atmosphere. Cell passages were performed every 2–3 days depending on the confluence of cells. First, film samples were cut into discs of 8 mm in diameter and pre-treated to avoid cell toxicity. Briefly, discs were dialyzed in 1 L of MilliQ water for 72 h into a dialyzing bag and then, freeze-dried. Dry discs were weighed and sterilized by UV radiation for 20 min. Subsequently, cells were seeded on top of them at a density of 5000 cells/well, and incubated at 37 °C for 4 and 7 days. Finally, discs were collected, washed with MilliQ, freeze-dried, and weighed. RM values were calculated as expressed by Equation (2). Three independent experiments, each of them with three replicates, were performed.

#### 2.4.11. Cytotoxicity Studies

Cytotoxicity studies were performed according to the ISO 10993-4:2009 [23] guideline for biological evaluation of medical devices. Firstly, film discs of 8 mm in diameter were pre-treated following the same procedure described in cellular degradation. Discs were sterilized exposing them to UV light for 20 min and dialyzed in 1 L of MilliQ water for 72 h. Then, they were transferred into culture medium for 24 h to reach osmotic equilibrium. Finally, pre-treated discs were divided into two groups to conduct direct or indirect cytotoxicity assays.

For direct cytotoxicity assay, L-929 fibroblasts were seeded onto a 24 well plate at a density of 35,000 cells/well and incubated overnight in order to allow cell attachment. Then, pre-treated discs were carefully placed into the wells. Cells incubated without any sample were used as positive control and cells incubated with 10% of DMSO (ATCC, Manassas, VA, USA) as negative control. After 48 h of incubation, discs were removed from the wells and viability was assessed. The different incubation time of direct and indirect assays was decided due to previous studies [24], where direct incubation needed more time to show the real cytotoxicity of the samples.

For indirect cytotoxicity assay, each disc was incubated with 0.5 mL of culture medium for 24 h to obtain the extracted medium. Meanwhile, cells were seeded on a 96 well plate at a density of 5000 cells/well and incubated overnight to allow cell attachment. Then, the medium was replaced with the extracted medium of the discs. Fresh medium was used as positive control and medium with 10% of DMSO as negative control. Cells were incubated for 24 h and then viability was evaluated.

In both cases, viability was assessed using the CCK-8 colorimetric assay (Cell Counting Kit-8, Sigma-Aldrich, Saint Louise, MO, USA). Briefly, 50 µL of the CCK8 reagent in the case of direct cytotoxicity and 10 µL in the case of indirect cytotoxicity were added to the cells, in each case 10% (*v*/*v*) of the volume of the wells. Cells were incubated for 4 h and then the absorbance of the wells was read at 450 nm, using 625 nm as a reference wavelength Plate Reader Inifinite M200 (Tecan, Barcelona, Spain). The absorbance values were directly proportional to the number of living cells in each well. Results were expressed as the percentage of living cells regarding the positive control. Three independent experiments, each of them with three replicates, were performed.

### 2.5. Statistical Analysis

For biological experiments, means were compared through one-way ANOVA. Based on the results of the Levene test for the homogeneity of variances, Bonferroni or Tamhane post-hoc analysis was applied. For non-normally distributed data, Mann–Whitney nonparametric analysis was applied. All the statistical computations were assessed using SPSS 25.0.

## 3. Results and Discussion

### 3.1. Physicochemical Properties

**Figure 2:**
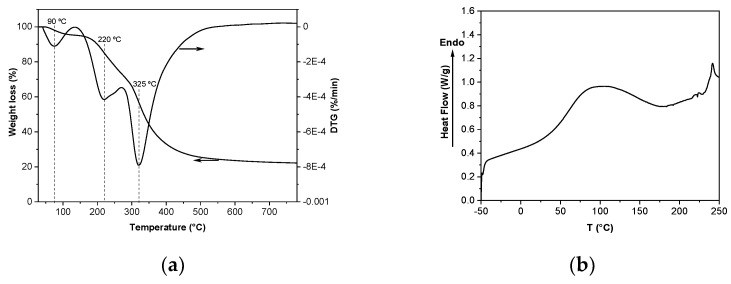
(**a**) Thermo-gravimetric analysis (TGA) and DTG curves, and (**b**) differential scanning calorimetry (DSC) curve of soy protein isolate (SPI) films.

The extrusion process used to mix, homogenize, and shape the SPI blend by forcing it through a specifically designed opening led to a continuous filament with no bubble at the extruder die. The process variable measured for this system was the specific mechanical energy (SME) and its value was 648 kJ/kg, with a stable torque of 18%. This SME value indicated the extent of molecular breakdown the material undergoes during the extrusion process [25]. This extrusion process led to the decrease in electrostatic repulsions and allowed intermolecular interactions and a new SPI network formation to occur. In particular, globular proteins, such as SPI, are triggered by thermal treatment and, thus, water and glycerol can enter the protein network and interact with protein chains by hydrogen bonding with easily accessible polar amino acids side chains, preventing protein–protein interactions and, thereby, leading to plasticization. Therefore, water and glycerol acted as a dispersion medium, influencing the extrudate viscosity and flowability. In order to assess the effect of the extrusion process, the thermal properties of SPI films were analyzed by TGA and DSC. As shown in Figure 2, the thermal behavior of SPI was not found to be affected by the extrusion process. The DTG curve showed three main stages (Figure 2a): the first stage around 90 °C was related to the moisture evaporation; the second stage was associated with glycerol evaporation [26] and appeared around 220 °C, a higher temperature than the boiling point of glycerol (182 °C), indicating the interactions between SPI and glycerol; and the third stage above 325 °C was attributed to the thermal degradation of SPI [27]. Therefore, the thermal stability of SPI was not affected by extrusion. Additionally, DSC analysis was carried out and a pronounced endothermic peak was observed around 100 °C (Figure 2b), corresponding to the denaturation of 7S globulins present in SPI, and a second one around 240 °C, related to the high molecular fraction of 11S globulins [28]. 

**Figure 3:**
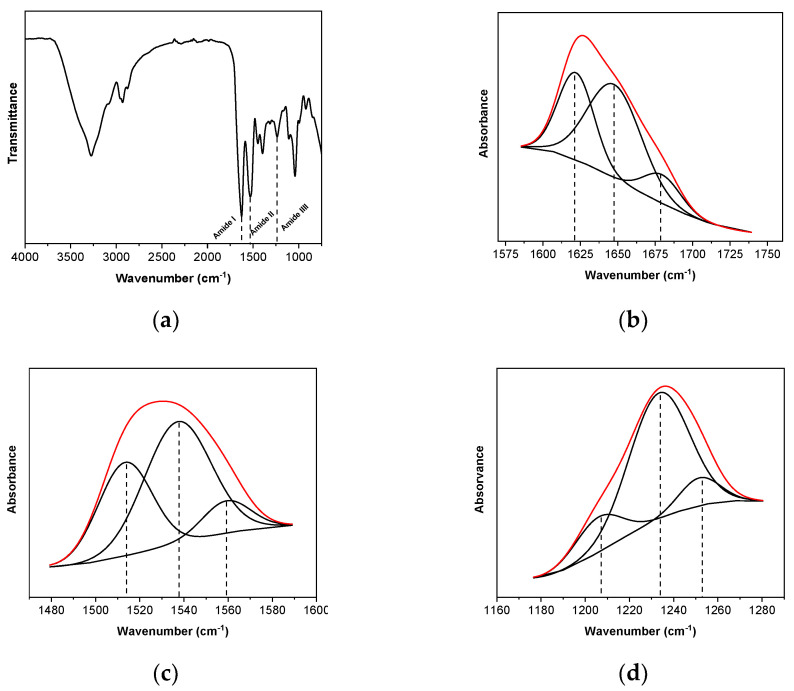
(**a**) FTIR spectrum and curve fitting of (**b**) amide I, (**c**) amide II, and (**d**) amide III of SPI films.

This thermal denaturation of the protein involved the disruption of intramolecular bonding and the unfolding and aggregation of protein molecules. Since the formation of a blend occurred upon application of denaturing conditions, the denatured protein may undergo partial refolding, thus regaining some secondary structure during the blending process. The extent of such refolding may affect the number of functional groups available for intermolecular interactions and, thus, the stability of the network formed. Regarding the processing method used, it is generally accepted that proteins are denatured during extrusion, so that the reactive unfolded protein chains can interact with each other and with glycerol, leading to the formation of a new network [18]. Since these intermolecular interactions formed in the SPI network offer great opportunities for the design of specific structures, FTIR analysis was carried out to assess them. FTIR spectrum of SPI films is shown in Figure 3a. The spectrum exhibited three characteristic bands common to proteins: amide I band at 1630 cm^−1^, associated with the carbonyl group; amide II band at 1530 cm^−1^, corresponding to N-H bending; and amide III band at 1230 cm^−1^, related to C-N stretching and N-H bending [29]. The broad band observed in the 3500–3000 cm^−1^ range is related to free and bound O-H and N-H groups, which are able to form hydrogen bonding. The band corresponding to amide I depends on the secondary structure of the protein backbone and it is the most commonly used band for the quantitative analysis of secondary structures. Therefore, a curve fitting treatment was carried out to estimate quantitatively the relative proportion of each component representing a type of secondary structure (Figure 3b–d). The derivative function was calculated to determine the number of components in the amide I region for the curve-fitting process. The areas of assigned amide I bands in the second derivative spectra correspond linearly to the number of different types of secondary structures present in the protein.

Table 1 shows the percentage of different types of secondary structures present in SPI films. Regarding amide I region, three bands can be distinguished. The band at 1649 cm^−1^ is observed in the FTIR spectra of most proteins, and it is assigned to the random coil, helical conformation, or both [30]. Additionally, the two bands at 1622 and 1678 cm^−1^ are assigned to β-sheet conformations [31]. On the one hand, the hydrogen bonding frequency dependence of the amide I vibration predominantly derives from the C=O group and, considering the central role hydrogen bonding plays in protein folding, results showed that interpeptide hydrogen bonding stabilized both α-helix/random coils and β-sheet secondary structures, which act as junction zones in the cohesion of film. On the other hand, the hydrogen bonding frequency dependence of the amide II vibration derives from N–H hydrogen bonding, which alters both the geometry and electronic density distribution. In relation to the band corresponding to amide II, the band at 1513 cm^−1^ was attributed to the vibrational modes of tyrosine side chains [32], which contribute at the interfaces of the β sheet regions, while the band at 1537 cm^−1^ was attributed to lysine side chains [32]. Concerning the band at 1559 cm^−1^, this was related to the carboxyl side groups of aspartic and glutamic acids present in SPI [33]. Finally, the amide III band is known to reveal some information on local stress and coupling effects of C–N stretching and H–N–C in plane bending modes and, thus, the deconvoluted amide III band region showed a band at 1206 cm^−1^ assigned to tyrosine/phenylalanine, a second band at 1233 cm^−1^ assigned to the random coil/helical conformation, and a third one related to β-sheets at 1252 cm^−1^ [34]. Due to these secondary structures, such as β-sheet and α-helix, in addition to the existence of peptide linkage, hydroxyl and carbonyl groups, SPI films show a structure of amino acids strongly interconnected through intra- and intermolecular hydrogen bonding that can provide films with electric properties.

### 3.2. Optical Properties

**Figure 4:**
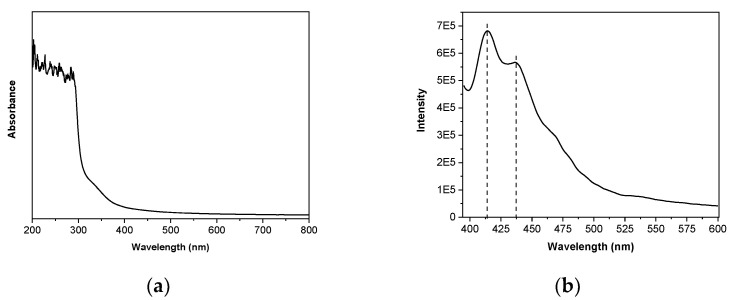
(**a**) UV-vis spectra and (**b**) fluorescence emission spectra (excitation at 370 nm) of SPI films.

Ultraviolet-visible (UV-vis) spectroscopy was used to measure barrier properties against UV-vis light. Figure 4a shows light absorbance values of SPI films. As can be seen, films showed excellent barrier properties against UV light in the range from 200 nm to 300 nm. The absorbance from 200 nm to 240 nm was associated with C=O, COOH, CONH_2_ groups in protein chains [35], and the absorbance at 250–280 nm was associated with the presence of sensitive chromophores in soy protein, such as tyrosine, phenylalanine and tryptophan [36]. The low absorbance value at 600 nm is associated with the transparency of SPI films and this is an indicator of the compatibility of the components used in the film-forming formulation [37], leading to homogeneous structures at the macroscopic scale.

In order to explore and elucidate the effect of the chromophores in SPI films, the fluorescence steady-state emission was measured and shown in Figure 4b. The fluorescence intensity of the SPI film was measured according to an excitation wavelength at 370 nm and emission wavelengths from 380 nm to 600 nm. The intensity of the peak at 413 nm corresponds to tryptophan, while the small shoulder at 437 nm corresponds to tyrosine and phenylalanine present in SPI [38]. It is known that pure tryptophan presents a fluorescence emission at 350 nm [39]; therefore, the red shift to 413 nm indicated that the tryptophan fluorescence emission was highly influenced by hydrogen bonding and other non-covalent interactions between soy protein and glycerol, as shown by FTIR results, causing a major exposition of tryptophan residues. Based on these results, the photo-induced conduction indicated an alteration of protein structure, conformation, and charge distribution, which directly affected electron transport properties.

### 3.3. X-ray Diffraction (XRD) and X-ray Photoelectron Spectroscopy (XPS)

**Figure 5:**
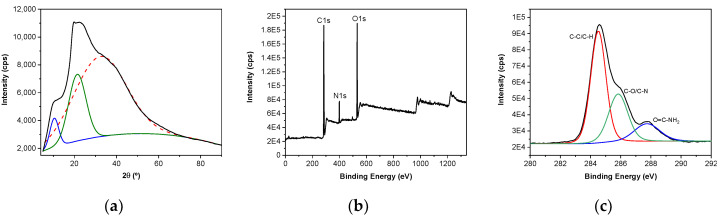
(**a**) X-ray diffraction (XRD) pattern, (**b**) X-ray photoelectron spectroscopy (XPS) survey data of SPI films, and (**c**) deconvolution of C1s spectrum.

The crystallinity degree of SPI films was determined by deconvolution from the X-ray diffraction (XRD) pattern. As can be seen by the broad peak in Figure 5a, SPI films are predominantly amorphous. The deconvolution of the XRD pattern showed a broad peak centered at 2θ of ~32.3°, associated with the amorphous structure of SPI, and the other two narrower peaks at 2θ of ~10.4° and ~21.4°, associated with the crystalline character of the films. The areas calculated for each of these peaks were 74.4%, 21.0% and 4.6% for the peaks centered at 2θ of ~32.3°,~21.4°, and ~10.4°, respectively, indicating a crystallinity degree of ~ 26%. The peak at 21.4° is associated with S-S bonds of cysteine in SPI films; these S-S bonds can tightly bind highly aligned β-sheets chains together, playing a relevant role in the electric dipole formation [40].

The XPS survey data analysis of SPI films (Figure 5b) showed the presence of three peaks, corresponding to C, N, and O. Additionally, the deconvolution of C1s spectrum showed other three peaks (Figure 5c): a dominant peak at 284.6 eV, attributed to C-C and C-H bonds; a peak at 285.8 eV, assigned to C-O/C-N; and the peak at 287.7 eV, assigned to O=C-NH_2_ bonds [41]. Furthermore, the high-resolution O1s spectra presented only one peak at 531.9 eV, attributed to O-C=O/O=C-N, whereas the high-resolution N1s spectra showed the presence of C=N/C-N at 399.9 eV. The small intensity of the peak related to N1s, compared to the ones corresponding to C1s and O1s, indicated the low exposure of amino groups towards the surface.

### 3.4. Film Microstructure and Mechanical and Electrical Properties

**Figure 6:**
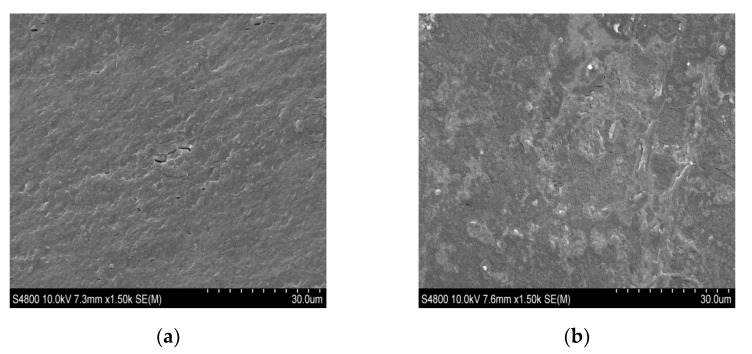
SEM images of (**a**) the cross-section and (**b**) the surface of SPI films at ×1.50 magnification.

**Figure 7:**
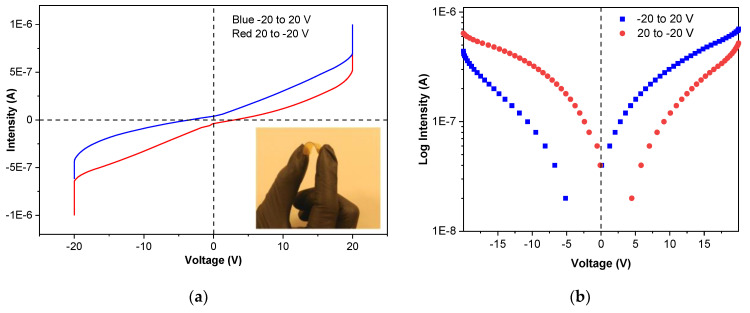
(**a**) Current intensity–voltage (I-V) curves (inset: SPI film flexibility) and (**b**) log I-V curves of SPI films.

In order to confirm the good compatibility among the components, previously suggested by optical results, scanning electron microscopy (SEM) analysis was carried out. The surface and cross-sections of SPI films are shown in Figure 6. As can be seen, films exhibited a homogeneous and compact structure (Figure 6a) with a rough surface (Figure 6b). Therefore, it can be assumed that during extrusion and film formation soy protein and glycerol were mixed without the formation of bubbles or holes. This evidenced significant molecular rearrangements within the polymer matrix, in which intermolecular interactions by hydrogen bonds occurred. This homogeneity of the film microstructure led to easy to handle films, as can be seen in the image in Figure 7a, with an elongation at a break value of 21.5 ± 1.7% and a tensile strength of 9.2 ± 0.5 MPa. Since mechanical properties are largely associated with the distribution and density of intramolecular and intermolecular interactions, which determined spatial structures, the flexibility of the films can also be related to the replacement of intramolecular interactions by intermolecular interactions by hydrogen bonds among SPI and glycerol, as shown above by FTIR results.

In order to analyze the effect of film structure on electrical conductivity, electron transport across SPI monolayer films was studied using solid-state protein-based molecular junctions with a current intensity-voltage (I-V) method, and results are shown in Figure 7a. As can be seen, SPI films showed a pinched hysteretic shape and, thus, behaved like memristors since I-V dependence in the range from −20 V to +20 V was nonlinear, the basis of functional electronic devices, as shown in Figure 7b. In relation to the electrical resistance and conductivity, the values obtained at room temperature were 1.011 × 10^3^ Ω m and 9.889 × 10^−4^ S/m, respectively, characteristic values of electrical semiconductor materials, such as silicon, and above the values of other well-known natural materials with satisfactory conductive properties [42].

The fundamental mechanism of electron transport (electron motion) via proteins is less understood than electron transfer (electron flow) due to the fact that electron transport measurements are not carried out in solution and, thus, there is no ionic charge in the medium surrounding the protein to screen charging as the electron moves across the protein. In this work, SPI films were measured between electronically conducting electrodes. This type of junction can be thought of as a donor–bridge–acceptor junction; the driving force of the transport is the electrical potential difference. The potential that the electron encounters when transported through the protein is affected by amino acid residues and their spatial arrangement. In this regard, protein structure can be understood at several levels: the primary structure, the linear sequence of amino acids held together by covalent peptide bonds, forming a polypeptide chain; the secondary structure, where the polypeptide chains form highly ordered three-dimensional features (α-helices and β-sheets) based on hydrogen bonding between peptide bonds; the tertiary structure, formed by additional secondary structure elements that undergo the necessary folding by specific interactions, including the formation of hydrogen bonds, and disulfide bonds giving rise to a compact structure [16]. The effect of secondary structure on electronic transport has been experimentally demonstrated with molecular junctions [43]. Therefore, the electron transport confirmed by I-V curves is in accordance with FTIR results, where the most prominent bands were related to the vibrations of peptide bonds at 1630 cm^−1^ and 1530 cm^−1^, corresponding to amide I and II vibrational modes, with other relevant bands assigned to vibrational modes of side groups, such as NH_2_ in-plane bending and C=C or C-N bonds, present in aromatic amino acid residues, such as tyrosine, phenylalanine, and tryptophan. These findings confirm that the side groups of amino acid residues play a relevant role in the inelastic part of the transport across the protein.

### 3.5. Water Uptake and Contact Angle

**Figure 8:**
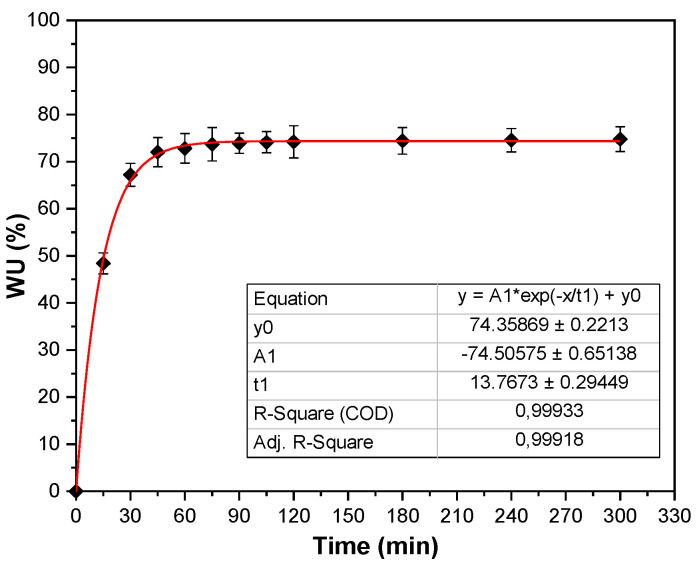
Water uptake of SPI films.

Results on water uptake showed that films were able to absorb the 72.1 ± 3.1% of their dry weight from 45 min onwards. No difference in water uptake was observed among time points, and the maximum value of 74.8 ± 2.6% was achieved at 120 min, as can be seen in Figure 8, where experimental values fit well (R = 0.999) with the empirical equation. The moderate swelling of SPI films is in accordance with the compact structure found by SEM analysis, in agreement with the intermolecular interactions between protein and glycerol indicated by FTIR results. This behavior suggests an enhancement of the conductivity properties since films could conduct both ionic and electronic currents [44], performance not provided by traditional metallic conductors.

**Figure 9:**
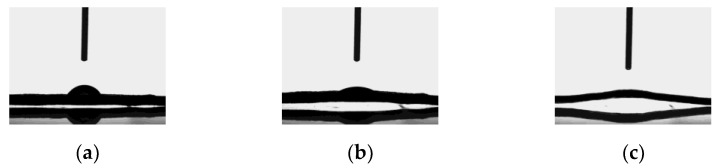
Contact angle of SPI films as a function of time: (**a**) t = 0 min, (**b**) t = 3 min, and (**c**) t = 5 min.

Regarding water contact angles, the values measured were 30.23 ± 0.76°, indicating that the SPI film surface was hydrophilic and, thus, those polar groups were orientated towards the surface. As can be seen in Figure 9a–c, SPI films absorbed the water droplet after 5 min. Since proteins are self-organized macromolecules with environment-sensitive secondary (α-helix and β-sheet), and tertiary (folding) structures, these results indicated that protein chains have the ability to rearrange when subjected to wet environments. Therefore, considering that a large number of functional interactions happen at surfaces, such as cell adhesion and growth, and that high hydrophilicity and water-retaining properties are vital for removing wound exudates and providing a moist environment for cell growth [45], degradation and biocompatibility studies were carried out.

### 3.6. Degradation and Biocompatibility Studies

**Figure 10:**
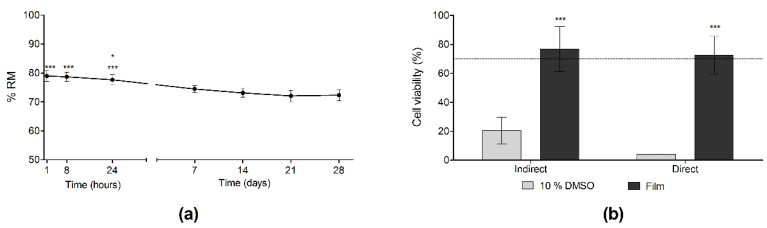
(**a**) Hydrolytic degradation. *** *p* < 0.001 comparing results at 1 h, 8 h and 24 h time points with the remaining time points, except from the comparison between 24 h and 7 days, when the statistical difference was lower (* *p* < 0.05), and (**b**) cell biocompatibility. *** *p* > 0.001 comparing the films with the negative control (10% DMSO).

SPI films showed a two-stage weight loss in an aqueous environment, as can be seen in Figure 10a. First, a very quick weight loss was observed, since RM values were 78.9 ± 1.7% in the first hour of immersion. Thereafter, weight loss was slower, since no significant differences were found until day 7, when RM values decreased to 74.5 ± 1.2%. From day 7 until the end of the study on day 28 (RM = 72.3 ± 1.9%), no significant difference was observed. Accordingly, only 28% of weight loss was observed, most of it due to glycerol loss.

Regarding enzymatic degradation, RM values were very similar at the three time points tested, about 72% of the dry weight. Comparing to the hydrolytic degradation, a significantly (** *p* < 0.001) faster weight loss was observed at 24 h in the samples incubated with the enzymatic solution. However, there was no difference in the assays at 4 and 7 days. Therefore, we can conclude that in both cases 28% of the weight was lost throughout the study; however, the weight loss occurred faster in an enzymatic media than in an aqueous media, highlighting that enzymes participate in film degradation.

In relation to cellular degradation, the RM values of SPI films incubated with cells at days 4 and 7 were 95.2 ± 1.4% and 94.2 ± 1.2%, respectively. The films were dialyzed in an aqueous media for 72 h prior to the study, in order to avoid possible cytotoxicity due to changes in pH and osmolarity and, thus, the initial weight loss of glycerol occurred during the pre-treatment and not during the cell degradation study. In this case, the initial weight of the films was lower than in the other degradation studies, since films were weighed after the pre-treatment, which explains the lower weight loss observed. At any rate, taking into account the weight loss that occurred during the pre-treatment, the real RM was 72.46 ± 9.6% and 73.24 ± 8.4% at days 4 and 7, respectively. This matches with the results obtained in the enzymatic degradation study, where the final weight loss was similar to the hydrolytic degradation study, but occurred faster.

Biocompatibility was evaluated to confirm the lack of cytotoxicity of SPI films. Before conducting the assays, films were dialyzed for 72 h, according to the results obtained in a preliminary study, where changes in pH and osmolarity were observed after the first 72 h of incubation with culture medium (data not shown). As can be seen in Figure 10b, the results obtained from both direct and indirect cytotoxicity assays showed that cell viability was above 70%. This confirmed that the type of assay did not affect cell viability results, which indicate the biocompatibility of SPI films according to the ISO 10993-5:2009 guidelines for the biological evaluation of medical devices [23]. 

## 4. Conclusions

In this work, SPI films for molecular electronic devices were modelled as one-dimensional solid-state conductors because it is known that the biological functions of proteins are dependent on their structure in a fundamental way. The SPI films obtained in this work by an industrial thermo-mechanical process, such as extrusion and compression, were transparent and homogeneous, with a compact structure and a rough surface. The intermolecular interactions between SPI and glycerol led to flexible films. Besides flexibility, SPI films showed a pinched hysteretic shaped intensity-voltage curve, indicating their performance as semiconductors. In contrast to the usually hard and dry electronic devices, SPI films were flexible and hydrophilic. This behavior of SPI films is of great relevance when films are intended to be put in close contact with the human body, where soft and flexible electronic devices are more suitable to fit the human body environment. In sum, easy to handle films were developed from natural raw materials, resulting in flexible and biocompatible films, which provide a promising strategy towards biocompatible organic electronics.

## Figures and Tables

**Table 1:** Secondary structure determination for amide I, II and III in SPI films.

FTIR Band	Amide I			Amide II			Amide III		
Wavenumber (cm^−1^)	1622	1649	1678	1513	1537	1559	1206	1233	1252
Area (%)	33.90	54.50	11.60	34.25	55.23	10.52	15.83	73.31	10.86

## Data Availability

The data presented in this study are available on request from the corresponding author.
